# Paternal involvement and early infant neurodevelopment: the mediation role of maternal parenting stress

**DOI:** 10.1186/s12887-016-0747-y

**Published:** 2016-12-12

**Authors:** Minjeong Kim, Su-Kyoung Kang, Bangsil Yee, So-Yeon Shim, Mira Chung

**Affiliations:** 1 Sesalmaul Research Center, Gachon University, Seongnamdae-ro 1342, Seongnam, 13120 Gyeonggi-do South Korea; 2 Department of Early Childhood Education, Gachon University, Seongnamdae-ro, 1342, Seongnam, Gyeonggi-do 13120 South Korea; 3 Department of Childcare & Education, Osan University, Cheonghak-ro 45, Osan, 18116 Gyeonggi-do South Korea; 4 Department of Pediatrics, School of Medicine, Ewha Womans University, 1071, Anyangcheon-ro, Yangcheon-gu, Seoul, 07985 South Korea

**Keywords:** Paternal involvement, Maternal parenting stress, Infant neurodevelopment, Mediation effect

## Abstract

**Background:**

Father–child interactions are associated with improved developmental outcomes among infants. However, to the best of our knowledge, no study has addressed the effects of paternal involvement on the neurodevelopment of infants who are less than 6 months of age, and no study has reported how maternal parenting stress mediates the relationship between paternal involvement and infant neurodevelopment during early infancy. This study investigates the direct and indirect relationship between paternal involvement and infant neurodevelopment at 3–4 months of age. The indirect relationship was assessed through the mediating factor of maternal parenting stress.

**Methods:**

The participants were recruited through the Sesalmaul Research Center’s website from April to June 2014. The final data included 255 mothers and their healthy infants, who were aged 3–4 months. The mothers reported paternal involvement and maternal parenting stress by using Korean Parenting Alliance Inventory (K-PAI) and Parenting Stress Index (PSI), respectively. Experts visited the participants’ homes to observe infant neurodevelopment, and completed a developmental examination using Korean version of the Ages and Stages Questionnaire II (K-ASQ II). A hierarchical multiple regression analysis was used for data analysis.

**Results:**

Infants’ mean ages were 106 days and girls accounted for 46.3%. The mean total scores (reference range) of the K-PAI, PSI, and the K-ASQ II were 55.5 (17–68), 45.8 (25–100), and 243.2 (0–300), respectively. Paternal involvement had a positive relationship with K-ASQ II scores (*β* = 0.29, *p* < 0.001) at 3–4 months of age, whereas maternal parenting stress was negatively related with K-ASQ II scores (*β* = −0.32, *p* < 0.001). Maternal parenting stress mediated the relationship between paternal involvement and early infant neurodevelopment (Z = 3.24, *p* < 0.001). A hierarchical multiple regression analysis showed that paternal involvement reduced maternal parenting stress (*β* = −0.25, *p* < 0.001), which led to positive infant outcomes (*β* = 0.23, *p* < 0.001).

**Conclusions:**

Paternal involvement is significantly associated with infant neurodevelopment during early infancy, and maternal parenting stress partially mediates that association. This result emphasizes the importance of fathers’ involvement and mothers’ parenting stress on early infant neurodevelopment.

## Background

Until now, studies on the effects of parenting have focused on maternal aspects [1–5]. For example, many studies have suggested that antenatal and postnatal maternal depression negatively affects children’s cognitive and psychosocial development [1–4]. Maternal parenting stress directly influences infant development, has a negative effect on parenting behavior, and delays stable attachment between parent and child [6, 7]. Recently, research regarding the role of the father in child development has increased. Fathers can play an integral role as attachment figures, and positive perceptions of fathering are consistently and significantly associated with caregiving activities and paternal warmth [8]. Fathers interact with their children differently from mothers, and healthy father–child interactions are associated with better neurodevelopmental outcomes for children later in life [9–11]. In fact, paternal involvement starts before a child is born. Several studies have suggested that internal paternal representations during pregnancy are associated with later child development [12, 13]. Paternal participation in caregiving and parenting changes according to infants’ age, and the paternal role is especially important in infancy [14]. Despite these paternal contributions to infant development, only a few studies have been conducted on the father–infant relationship during early infancy [15, 16]. Furthermore, no study has addressed the effects of paternal involvement on the infant neurodevelopment at 3–4 months of age. Traditionally, mothers have taken more responsibility for infant care than fathers have; therefore, it is important to control for maternal aspects, such as maternal parenting stress, when investigating how paternal involvement affects infant development. However, to the best of our knowledge, no study has investigated the presence of a mediation mechanism by showing that the effects of paternal involvement on early infant neurodevelopment are mediated through maternal parenting stress.

The present study investigates the direct and indirect relationship between paternal involvement and infant neurodevelopment at 3–4 months of age. Indirect relationship was assessed through the mediating factor of maternal parenting stress.

## Methods

### Recruitment

The present study was conducted from April to June 2014. A total of 456 mothers and their healthy infants, who were aged 3–4 months, were recruited through the website of Sesalmaul Research Center at Gachon University. They were contacted, and the study procedure and ethics compliance statements were explained to them before data collection. Among enrolled participants, only unemployed and married mothers, and their infants, aged 3–4 months and without medical problems, were included for this study. Participants who did not give informed consent, gave unreliable responses in which too many items were unanswered, or who gave the same type of answer throughout were excluded. These data accounted for 5% or less of the sample. They were statistically excluded by listwise deletion. Infants with congenital malformations, brain abnormalities, asphyxia, and prematurity (<36 gestational weeks at birth) were excluded; they accounted for approximately 1% of enrolled infants. Finally, 255 mothers and their healthy infants, who were aged 3–4 months, were included in this study. The mothers reported paternal parenting involvement and maternal parenting stress by using the Korean Parenting Alliance Inventory (K-PAI) and the Parenting Stress Index (PSI), respectively. Experts visited the participants’ homes to assess infant neurodevelopment at 3–4 months of age. These experts were composed of professionals with a Master’s degree in early childhood education and experience as teachers in kindergarten or childcare centers. They were trained 4 times in the use of the study instruments (3 h each time) at Sesalmaul Research Center. The experts observed each infant for 90 min and completed a neurodevelopmental checklist with the mothers, using the Korean version of the Ages and Stages Questionnaire II (K-ASQ II) for 4-month-old infants.

### Measures

#### The Korean Parenting Alliance Inventory (K-PAI)

The PAI [17] and the Father Caretaking Inventory [18] were modified to create the K-PAI [19]. The present study used the K­PAI to assess paternal involvement in parenting. This measure consists of three subscales and seventeen items. Caretaking activity comprises 10 items (e.g., “My husband helps me by soothing the baby if he or she cries at night”), 2 items are related to shared philosophy and parenting perceptions (e.g., “My husband and I share the same values about child rearing”), and emotional appraisal of a spouse’s parenting includes 5 items (e.g., “It is very helpful to talk to my husband about our child”). The mothers responded to each item using a 4-point Likert-style scale that ranged from 1 to 4 (1: strongly disagree, 2: disagree, 3: agree, 4: strongly agree).

#### The Parenting Stress Index (PSI)

The PSI [20] consists of three subscales, namely, parental distress (10 items; e.g., “trapped by parenting”), dysfunctional parent–child interactions (8 items; e.g., “rarely makes me feel good”), and difficult child (7 items; e.g., “smiles less”). The mothers responded to each item using a 4-point Likert-style scale based on their agreement with each statement (1: strongly disagree, 2: disagree, 3: agree, 4: strongly agree).

#### The Korean version of Ages and Stages Questionnaire II (K-ASQ II)

The ASQ is a parent-completed, child-developmental screening test with 5 domains (communication, gross motor, fine motor, problem-solving, and personal/social), each with 6 items. The items are written at the fourth- to sixth-grade reading level and can be administered in the form of an interview to parents with low literacy levels. The ASQ II was standardized to use on children aged 4 to 60 months. In a recent multinational trial involving 18 countries in Asia, Africa, Europe, and North- and South-America, sensitivity was 88% and specificity was 82.5% [21]. The Korean version of the ASQ II for 4-month-old infants was used in this study. The K-ASQ II has been validated and standardized for Korean children, based on the ASQ II [22]. The ASQ and K-ASQ questionnaires are valid for populations ± one month of the target age [21–23]. The K-ASQ II comprises 5 domains and 30 items; the domains include the following items: communication (e.g., “Does your baby chuckle softly?”), gross motor skills (e.g., “While on his/her back, does your baby turn his/her head to both sides?), fine motor skills (e.g., “Does your baby hold his/her hands open or partly open?”), problem solving (e.g., “When you move a toy slowly from side to side in front of his/her face, does your baby follow the toy with his/her eyes, sometimes turning his/her head?”), and personal/social skills (e.g., “Does your baby watch his/her hands?”). Each domain consisted of 6 items, and possible responses for each item were “yes” (10 points), “sometimes” (5 points), and “no” (0 points). When an infant scores 2 SD below the mean on at least one domain, immediate referral for further assessment was advised. The 2 SD scores for 4-month-old infants correspond to 35, 28, 18, 30, and 25 in communication, gross motor, fine motor, problem-solving, and personal/social domains, respectively [23].

### Approach to analysis

The data were analyzed using SPSS 21.0. A Pearson’s correlation analysis tested the direct relationships between infant neurodevelopment and either paternal involvement or maternal parenting stress. To adjust for demographic and SES variables for those analyses, multivariate analysis was conducted. To test the mediation effect of maternal stress, a hierarchical multiple regression analysis was conducted based on Baron and Kenny’s method [24]. The significance of the mediation effect was tested through the Sobel test equation.

## Results

### Population characteristics

Table 1 shows the demographic characteristics of the participants. The infants’ mean age was 106 days. The mean ages of fathers and mothers were about 34 and 32 years, respectively, and their average level of education was a college degree. First-born infants accounted for 91.4% of the study infants. Most of the participants had family incomes that were approximately equal to South Korea’s national average. In South Korea, the average monthly family income is 2900 US dollars, according to the 2015 annual report by Statistics Korea (http://kosis.kr).

**Table 1:** Demographic data of study population (*n* = 255)

Variables	*n* (%) unless stated
Infant’s age (days)^a^	106.0 (10.6)
Girl	118 (46.3)
First-born infant	233 (91.4)
Vaginal delivery	170 (92.5)
Maternal age (years)^a^	31.8 (3.1)
Paternal age (years)^a^	33.8 (3.3)
Maternal level of education	
High school	19 (7.5)
College	194 (76.1)
Graduate school	42 (16.5)
Paternal level of educational	
High school	14 (5.5)
College	206 (80.8)
Graduate school	35 (13.7)
Monthly family income (US dollars)	
Less than 2000	24 (9.4)
2000-4000	176 (69.0)
More than 4000	55 (21.6)

^a^Values are expressed as mean (± standard deviation)

### Scores of measures

The means and standard deviations of the data for the K-PAI, PSI, and the K-ASQ II are shown in Table 2. The mean total scores of the K-PAI, PSI, and the K-ASQ II are 55.5, 45.8, and 243.2, respectively. The alpha coefficients for the scales were 0.63–0.89 for the K-PAI, 0.76–0.87 for the PSI, and 0.84 for the K-ASQ II. Infants with suggestive delayed development in communication, gross motor, fine motor, problem-solving, and personal/social domains accounted for 6.7% (17 infants), 4.3% (11 infants), 2.0% (5 infants), 5.9% (15 infants), and 4.3% (11 infants) of the sample, respectively. For all three measures, the skewness was −1.244 to 1.117, that is, in the range of 3 (absolute value); the kurtosis was −0.695 to 1.259, that is, in the range of 8 (absolute value). The data met the normality of distribution.

**Table 2:** The scores of measures (*n* = 255)

Variables (Reference range)	Mean (SD) scores
Korean Parenting Alliance Inventory (K-PAI)^a^	
Caretaking activity (10–40)	32.1 (5.0)
Shared philosophy and perceptions of parenting (2–8)	6.8 (1.2)
Emotional appraisal of spouse’s parenting (5–20)	16.6 (2.3)
Total scores (17–68)	55.5 (7.4)
Parenting Stress Index (PSI)^b^	
Parental distress (10–40)	14.0 (3.8)
Difficult child (7–28)	13.4 (3.1)
Parent–child dysfunctional interaction (8–32)	18.5 (3.0)
Total scores (25–100)	45.8 (7.3)
Korean-Ages and Stages Questionnaire II (K-ASQ II)^c^	
Communication (0–60)	51.6 (8.6)
Gross motor (0–60)	49.5 (11.0)
Fine motor (0–60)	44.0 (11.3)
Problem-solving (0–60)	49.0 (10.0)
Personal/social (0–60)	49.1 (10.2)
Total scores (0–300)	243.2 (37.4)

Values are expressed as mean (± standard deviation). The higher scores mean the better level of paternal involvement^a^, more maternal stress^b^, and better neurodevelopment of infants^c^

### Adjusting demographic/socioeconomic background, the effects of paternal involvement and maternal parenting stress on infant neurodevelopment

Table 3 shows that the multivariate analysis results for total scores on the K-ASQ II, adjusting for demographic variables and the socioeconomic status (SES) of the family. In univariate analysis, there was no statistical significance between each SES and demographic variable and the K-ASQ total scores (the range of *F* = 0.021–0.928 and the range of *p* = 0.117–0.886; data are not shown in table). Paternal involvement and maternal parenting stress are associated with the total score on the K-ASQ II after adjusting for demographic variables and SES. Neither the demographic data nor SES, such as parents’ education and family income, were correlated with the total scores on the K-ASQ II (Table 3).

**Table 3:** Multiple regression analysis for the K-ASQ II total score adjusting for demographic and socioeconomic background (*n* = 255)

Independent variables	K-ASQ II total	
	Coefficient (SE)	*p*-value
Infant’s sex (girl)	0.06 (0.15)	0.318
First-born infant	−0.06 (0.03)	0.859
Maternal age (years)	0.06 (0.03)	0.422
Paternal age (years)	0.06 (0.17)	0.432
Maternal level of education^a^	−0.10 (0.16)	0.340
Paternal level of education^a^	0.08 (0.07)	0.125
Monthly family income^b^	0.01 (0.26)	0.210
Paternal involvement (K-PAI total score)	0.23 (0.18)	<0.001
Maternal parenting stress (PSI total score)	−0.26 (0.25)	<0.001

^a^Variables include high school, college, and graduate school. ^b^Variable includes coding numbers representing less than 2000, 2000-4000, and more than 4000 US dollars

### Correlation among paternal involvement or maternal parenting stress, and infant neurodevelopment

Table 4 shows that the total scores on the K-PAI were positively correlated with the total scores of the K-ASQ II (*r = 0.29, p < 0.001*). All subscales of the K-PAI, such as caretaking activity, shared philosophy and perception, and emotional appraisal were correlated with the total scores of the K-ASQ II (*p <* 0.001, *p =* 0.003, and *p <* 0.001, respectively). In Table 4, the subscales of maternal PSI, such as parenting distress, difficult child, and mother–child dysfunctional interaction were negatively correlated with the total scores of the K-ASQ II (*p <* 0.001, *p =* 0.001, and *p =* 0.001, respectively).

**Table 4:** Pearson’s correlation coefficient between the K-ASQ II total score and either the K-PAI or PSI scores (*n* = 255)

Variables	Coefficient	*p*-value
Paternal involvement (K-PAI)		
Caretaking activity	0.29	<0.001
Shared philosophy & perception	0.18	0.003
Emotional appraisal	0.26	<0.001
Total scores	0.29	<0.001
Maternal parenting stress (PSI)		
Parental distress	−0.29	<0.001
Difficult child	−0.20	0.001
Mother-child dysfunctional interaction	−0.21	0.001
Total scores	−0.32	<0.001

### The mediation effect of maternal parenting stress: the direct and indirect influences of paternal involvement to infant neurodevelopment

**Fig. 1:**
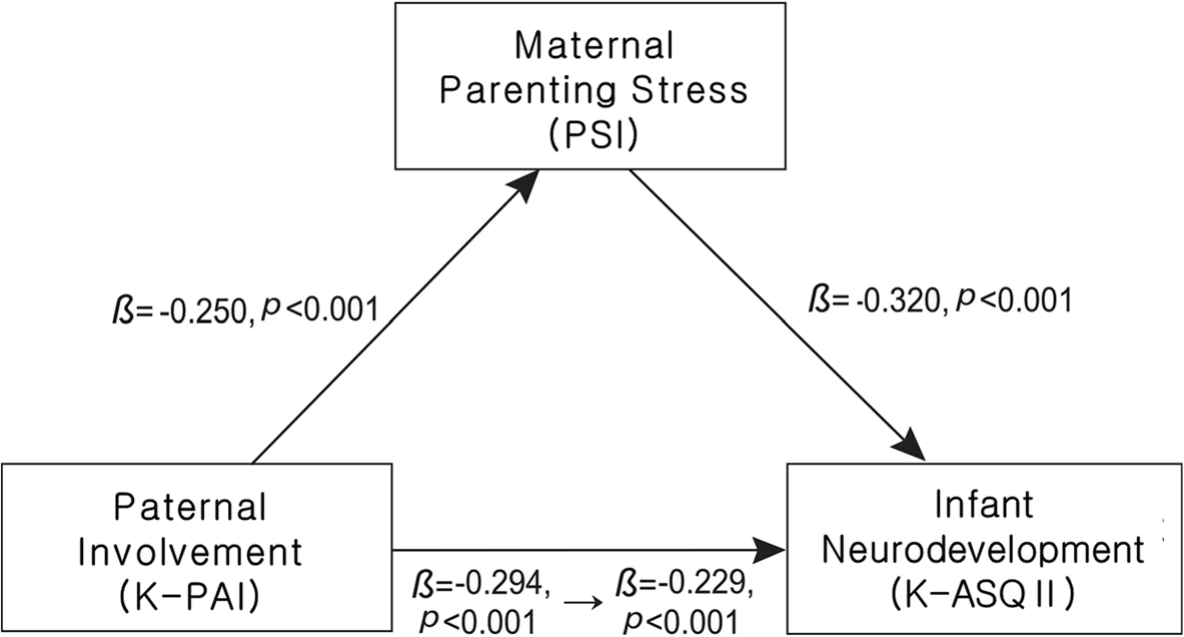
Mediation role of maternal parenting stress on infant neurodevelopment based on hierarchical multiple regression. Maternal parenting stress negatively affects infant neurodevelopment (*β* = −0.320, *p* < 0.001). Paternal involvement can reduce maternal parenting stress (*β* = −0.250, *p* < 0.001), resulting in having a positive effect on infant neurodevelopment (*β* = 0.229, *p* < 0.001). However, the direct effect of paternal involvement on infant neurodevelopment is stronger (*β* = 0.294, *p* < 0.001) than the indirect effect of paternal involvement thorough mediating factor of maternal parenting stress

Table 5 and Fig. 1 are the result of the Baron and Kenny’s method to present the direct and indirect effects of paternal involvement on infant neurodevelopment at 3–4 months of age. In Table 5, step 1 revealed that paternal involvement directly influences infant neurodevelopment (*β* = 0.29, *p* < 0.001). Step 2 showed that maternal parenting stress is negatively related to infant neurodevelopment (*β* = −0.32, *p* < 0.001). In step 3, the data relating to maternal PSI and paternal K-PAI were analyzed together to investigate whether the relation between K-PAI and K-ASQ II total scores is mediated by maternal PSI. If that significant relation was disappeared by maternal PSI mediation, then it could be assumed that the effect of paternal involvement on infant neurodevelopment was completely mediated by maternal parenting stress. Step 3 results show that the positive effect of paternal involvement on infant neurodevelopment decreased, but remained significant (*β* = 0.23, *p <* 0.001). As also shown in Fig. 1, this means that maternal parenting stress partially mediated the relation between paternal involvement and infant neurodevelopment (Z = 3.24, *p* < 0.001).

**Table 5:** The mediation effect of paternal involvement between maternal parenting stress and K-ASQ II using the Baron and Kenny’s method (*n* = 255)

Step	Independent variables	Dependent variable	*B*	*SE*	*β*	*t*	*p*
1	Paternal involvement	K-ASQ II	0.83	0.17	0.29	4.90	<0.001
2	Maternal parenting stress	K-ASQ II	−1.35	0.25	−0.32	−5.37	<0.001
3	Paternal involvement	K-ASQ II	0.65	0.17	0.23	3.82	<0.001
	Maternal parenting stress		−1.11	0.25	−0.26	−4.39	<0.001

## Discussion

This is the first study to demonstrate that paternal involvement is associated with infant neurodevelopment at 3–4 months of age. This study found that fathers directly affect infant neurodevelopment through caretaking activities, such as changing diapers, feeding, or dressing, and through emotionally supporting their partners. The present study showed that fathers also affect their infants indirectly through their influence on maternal parenting stress. A hierarchical multiple regression model revealed the relationships among paternal involvement, maternal parenting stress, and infant neurodevelopment. These three variables were moderately related to each other. Several possible explanations exist for the indirect association of fathers with infant neurodevelopment through mediation by maternal parenting stress. First, fathers who are sensitive to their infants may have a more positive relationship with their partners, which reduces maternal stress and enables a positive mother–infant relationship [16, 25]. The K-PAI subscales, such as shared philosophy and perception and emotional appraisal of a spouse’s parenting, reflect the relationship between the fathers and their partners [26]. The strong correlation between these subscales and infant neurodevelopment revealed the importance of the father–mother relationship to infant neurodevelopment. Second, paternal involvement in caretaking activities likely reduces the maternal burden. Third, the gatekeeper role of a mother should be considered: some mothers who have negative relationships with their partners tend to prevent them from being involved in infant care [27]. Postnatal maternal stress or depression is a major cause of negative outcomes for infants [2–4]. A consistent link has been established between postpartum depression and maternal parenting stress [28]. Our results showed that the PSI subscale, “parenting distress,” was correlated with infant neurodevelopment. These facts emphasize the importance of finding ways to reduce maternal distress during the early postnatal period, and the present study suggests that paternal involvement in parenting is one way of accomplishing this. Our finding that paternal involvement matters is consistent with previous studies that show that it benefits the neurodevelopmental outcomes of children [8–13, 15, 29]. Ramchandami et al. found that disengaged interactions between fathers and their 3-month-old infants predict behavioral problems at 1 year of age [15]. Sarkadi et al. concluded in their systematic review that paternal involvement positively affects the overall outcomes of children [10]. However, these studies estimated child neurodevelopment after 1 year of age. The present study reveals that infant neurodevelopment benefits from paternal involvement even at 3–4 months of age, although additional studies are necessary to confirm whether this benefit continues into later childhood.

Several studies have shown that SES, such as paternal level of education, income, and marital status, are associated with child outcomes [11, 16, 30, 31]. Surprisingly, the Korean population of college graduates is approximately 80%, similar to the present study. However, graduation from college does not seem to guarantee higher SES in Korea. This study highlights the effects of paternal involvement in infant neurodevelopment by showing multivariate analysis results, adjusting for SES. The present study has significant strength. The sample size was relatively large, compared with previous studies [9, 12, 15, 16], and each infant’s neurodevelopment was observed and measured by experts through home visits. However, several limitations of this study should be considered. Because we selected only unemployed and married mothers and over 90% of study infants are first-born infants, our results may not be generalizable to the entire Korean population. We attempted to clearly demonstrate the effects of paternal involvement by controlling for the maternal variable, because maternal employment affects maternal parenting stress [32] and a child’s neurodevelopmental outcomes [33, 34]. Our results cannot be applied to infants with specific medical issues. However, many other studies have addressed infants with specific conditions [26, 31, 35–38]. Another limitation is that the mothers provided the data regarding paternal involvement and maternal parenting stress. In a previous study, paternal involvement was overestimated when fathers answered, whereas it was underestimated when rated by mothers [39]. We thought that mothers’ assessment of fathers’ involvement corresponded to the goal of this study, in its investigation of the influence of the paternal involvement on mothers’ parenting stress. Although questionnaires are a less objective method than video-based assessment is, they remain the most commonly utilized assessments for this type of study. Furthermore, previous studies have suggested that the questionnaires that were used in the present study can predict child outcomes [28, 36]. The infant neurodevelopment assessments were conducted at an early age using the K-ASQ II. Follow-up study for this study population may be required because neurodevelopmental assessment for infants aged 3–4 months is limited. Although the Bayley Scales of infant and toddler development, third edition is a more well-known developmental screener, the Korean version thereof has not yet been standardized. A multidisciplinary panel of specialists in child neurology, pediatrics, psychology, and psychiatry endorsed the ASQ as a recommended screening tool for early identification of developmental disorders among children aged with age-range of 4–60 months [40, 41]. The ASQ has been used for developmental monitoring in several large-scale, pediatric research studies [21, 40–42]. Similar to the ASQ II, the K-ASQ II is widely used and considered to be a valid measure of early infant neurodevelopment in Korea [22, 37].

## Conclusions

This is the first study to show that paternal involvement is associated with infant neurodevelopment, even during early infancy, although neurodevelopmental assessment at the age of 3–4 months is limited. Paternal caretaking activities and emotional support for a partner are important factors that are directly related to infant neurodevelopment. In addition, the present study demonstrates the interactions among paternal involvement, maternal parenting stress, and infant neurodevelopment by using a well-controlled statistical method. Paternal involvement, specifically father’s caretaking activities and emotional support towards their spouse can reduce maternal parenting stress, which leads to positive infant outcomes. The present study contributes to the literature by emphasizing that direct parental involvement and the interactions between fathers and mothers are critical factors to address during early infant neurodevelopment.

## Abbreviations

K-ASQ II: Korean-Ages and Stages Questionnaire II
K-PAI: Korean Parenting Alliance Inventory
PSI: Parenting Stress Index
SES: Socioeconomic status
